# Patient reported outcome measures in children and adolescents with type 1 diabetes using advanced hybrid closed loop insulin delivery

**DOI:** 10.3389/fendo.2022.967725

**Published:** 2022-08-19

**Authors:** Ana Gianini, Jana Suklan, Brigita Skela-Savič, Simona Klemencic, Tadej Battelino, Klemen Dovc, Nataša Bratina

**Affiliations:** ^1^ Department of Pediatric Endocrinology, Diabetes and Metabolic Diseases, University Children’s Hospital, University Medical Centre Ljubljana, Ljubljana, Slovenia; ^2^ Slovenia and Faculty of Medicine, University of Ljubljana, Ljubljana, Slovenia; ^3^ NIHR Newcastle In Vitro Diagnostics Co-operative, Faculty of Medical Sciences, Translational and Clinical Research Institute, Newcastle University, Newcastle upon Tyne, United Kingdom; ^4^ Department for Masters and Phd in Health Care Science, Angela Boškin Faculty of Health Care, Jesenice, Slovenia

**Keywords:** type 1 diabetes, advanced hybrid closed-loop, patient report outcome measure (PRO), HbA1c, time in range

## Abstract

**Purpose:**

To determine the impact of advanced hybrid closed - loop (AHCL) insulin delivery on quality of life, metabolic control and time in range (TIR) in youth with type 1 diabetes mellitus (T1DM).

**Methods:**

Twenty-four children and adolescents with T1DM (14 female) aged of 10 to 18 years participated in the study. Mixed methods study design was implemented. Quantitative part of the study was conducted as a longitudinal crossover study with data collection before and at the end of AHCL use. Qualitative data were obtained with modeled interviews of four focus groups before and the end of the period. Clinical data were collected from the electronic medical records.

**Results:**

The use of AHCL significantly improved the quality of life in terms of decreased fear of hypoglycemia (p<0.001), decrease in diabetes-related emotional distress (p<0.001), and increased wellbeing (p=0.003). The mean A1C decreased from 8.55 ± 1.34% (69.9 ± 12.3 mmol/mol) to 7.73 ± 0.42 (61.1 ± 2.2 mmol/mol) (p=0.002) at the end of the study. Mean TIR was 68.22% (± 13.89) before and 78.26 (± 6.29) % (p<0.001) at the end of the study.

**Conclusion:**

The use of advanced hybrid closed loop significantly improved the quality of life and metabolic control in children and adolescents with T1DM.

## Introduction

Type 1 diabetes (T1DM) is one of the most common chronic conditions in children and adolescents all over the world with an increasing incidence of approximately 3.4% per year in Europe (1). The principal goal in the management of T1DM is to maintain blood glucose levels as close to normal as possible with the aim of avoiding or delaying disease-related micro and macrovascular complications, which represent the major cause of morbidity and mortality in developed societies (2, 3). The majority of people with T1DM does not achieve glycated hemoglobin (A1C) below 7% (53 mmol/mol) (4), as suggested by all major guidelines (5). The use of technology in the management of T1DM is becoming the predominant component of care, often first with a continuous glucose monitor and an insulin pump as needed, and most recently with systems combining the two into an automated closed-loop insulin delivery system (6, 7). The use of closed-loop systems as an acceptable therapeutic modality for management of T1DM has been the focus of many recent studies. There is a growing body of evidence that advanced hybrid closed loop (AHCL) systems improve glycemic control irrespective of baseline A1C, while simultaneously decrease the rates of hypoglycemic events (8, 9). Glycemic control using AHCL is maintained particularly well overnight, which is of great importance to parents of children with T1DM who experience significant fear of undetected hypoglycemia and hyperglycemia during the sleep (10, 11). AHCL insulin delivery, characterized by automated insulin delivery apart from prandial boluses, now represents routine clinical care for people with T1DM (12–17). The most recent data clearly confirm the ability of the AHCL insulin delivery systems to safely achieve a significant improvement of glucose control in T1DM (18).

In our study we aimed at determining the impact of AHCL usage on patient reported outcome measures.

## Methods

We utilized a mixed methods research design using a quantitative and a qualitative part. Data were obtained from a cohort longitudinal crossover study in which same participants were investigated at two different points in time.

Study participants were identified from the Slovenian National Diabetes Registry and invited to participate in the study through outpatient clinic, National Diabetes Society webpage, and social media. Study was conducted at the University Children’s Hospital in Ljubljana, Slovenia, which is a national center for childhood diabetes (19).

Ethical approval was obtained from the Slovenian National Medical Ethics Committee. All participants and their parent or legal guardian signed informed consent prior to the enrolment in the study.

### Inclusion/exclusion criteria

Inclusion criteria: participants were 10 - 18 years old (inclusive), had clinical diagnosis of T1DM for at least 6 months, and were using an insulin pump for at least 3 months. Exclusion criteria included concomitant diseases that could influence metabolic control or compromise a participant’s safety.

### Procedures

Data were obtained first before the start of AHCL usage, when conventional therapy was used, and again after 4 months at the end of the study. At baseline (conventional therapy), one participant was using insulin pump without continuous glucose monitoring (CGM), nine participants were using insulin pump with intermittently scanned CGM, four were using predictive low glucose suspend system (PLGS) and 10 were using first generation hybrid closed-loop device (HCL). During the study, participants were using a modified investigational version of Minimed 780G (Medtronic, USA) (AHCL). Compared to first generation HCL, AHCL includes several algorithmic developments and features with the aim of remaining in closed-loop for longer periods of time without compromising safety, including automated correction boluses.

Upon entry into the study, all participants began to use AHCL and received structured education. Validated questionnaires were applied, and downloads from their previous sensor and insulin pump were obtained along with the clinical data from the electronic medical record.

The participants answered 3 validated questionnaires: C-HFS – Children, Hypoglycemia Fear Survey; PAID Problem Areas in Diabetes Scale; PAID-5 Problem Areas in Diabetes Scale—Five-item short form; WHO-5 Five Item Measure of Wellbeing.

The C-HFS is validated for youth to assess their Fear of Hypoglycemia (FoH). It consists of two subscales: a Behavior subscale (11-items) and a Worry subscale (15-items). The Behavior subscale measures the *behaviors* of children and adolescents to prevent hypoglycemic episodes and their consequences, similarly the Worry subscale measures *concerns* related to hypoglycemia and its negative consequences. Items are rated on a 5-point Likert scale (1 = “never” to 5 = “almost always”). Questionnaire scores are computed as a total score divided by the total number of items to obtain an item mean score. Higher scores indicate higher FoH.

Problem Areas in Diabetes (PAID) is another well-validated and commonly used 20 items questionnaire used for measuring diabetes-related emotional distress, covering negative emotional problems. Items are rated on a 5-point Likert scale (0 = “not a problem” to 4 = “serious problem”). Questionnaire scores are computed as a total sum is multiplied by 1.25 to produce a final possible score of 0–100, with higher scores indicating greater diabetes-related emotional distress.

The World Health Organization–Five Well-Being Index (WHO-5) is a short self-report measure of subjective well-being with good psychometric properties. The scale measures the degree to which the positive feelings were present in the last 2 weeks is scored on 6-point Likert scale ranging from 0 (not present) to 5 (constantly present). The total raw score, ranging from 0 to 25, is multiplied by 4 to give the final score, 0 (worst thinkable well-being) to 100 (best thinkable well-being). A score < 50 suggests poor emotional well-being, while a score ≤ 28 is indicative of depression.

In the qualitative part of the study, we conducted focus groups with modelled interviews in order to obtain data that could not be obtained with the quantitative methods. Focus groups were conducted twice: first at study entry when conventional therapy was used, and second at the end of the study after 4 months of AHCL use. Children and adolescents were separated into four focus groups. The facilitator used a semi-structured questionnaire to present the main topic for discussion and a set of ‘ground rules’ as well as relevant ethical issues around data capture, storage and dissemination. The reliability of the study was ensured by the sufficient number of focus groups, and the number of participants in each group. A focus groups discussions continued until data saturation was reached (20). We audio-recorded the focus group’s conversations and performed transcripts of the recordings.

### Statistical analyses

Cronbach alpha was used to calculate the scale reliability (internal consistency) of the measurement scale. Paired sample two-tailed t-test with Holm – Bonferroni sequential multiplicity correction (21) was used to compare mean values from the beginning of the study with mean values from the end of the study adjusting for multiple comparisons. Since we conducted 9 comparisons, we are only able to reject the null hypothesis of each comparison if it has a p-value less than 0.006. The analysis of the data was done using IBM SPSS Statistics for Windows 27.0.

## Results

In total, 24 participants (14 females) with T1DM on insulin pump were enrolled and competed the study (mean age 14.5 ± 1.7 years, T1DM duration mean 7.2 ± 3.7 years) (**Table 1**), among them 17 school-aged children (10-14 years) and 7 adolescents (15-18 years). The average use of the insulin pump was 6.3 ± 4.2 years.

**Table 1:** Baseline characteristics.

N (%), Mean (SD)		
Age		14.5 ± 1.7
Comorbidities (Hashimoto, celiac)		5 (20.8%)
Diabetes duration (years)		7.2 ± 3.7
Insulin pump usage (years)		6.3 ± 4.2

Data presented as mean ± standard deviation or count (percentage).

### Patient-reported outcome measures (PROMs)

Patient-reported outcome measures were gathered using 3 validated, standardized questionnaires (C-HFS, PAID, WHO-5). All participants (n=24) included in the study completed all 3 questionnaires at the beginning and at the end of the study. Data suggest acceptable internal consistency of the sample with alpha values ranging from 0.866 to 0.925 (**Table 2**).

**Table 2:** Scale score reliability.

	Cronbach’s α			Range
Measure	Beginning of the study (n=24)	End of the study (n=24)	Number of variables	
C-HFS Total	0.925	0.889	25	25-125
PAID	0.908	0.888	20	0-100
WHO-5	0.906	0.866	5	0-100

C-HFS – Children, Hypoglycemia Fear Survey.

#### Fear of hypoglycemia

The combined results (behavior and worry subscale) showed a statistically significant decrease of fear of hypoglycemia with the use of AHCL. Statistical significance was calculated for all three groups (behavior, worry subscale and combined results) (**Table 3**).

**Table 3:** Comparison of average Behavior and Worry, C-HFS total score with a post - hoc analysis for participants with A1C < 7% (< 53 mmol/mol) versus A1C ≥ 7% (≥ 53 mmol/l).

		C-HFS Behavior subscale			C-HFS Worry subscale		C-HFS Total	
		Mean (SD)			Mean (SD)		Mean (SD)	
Beginning of the study (n=24)		29.25 ± 5.9			31.25 ± 13.5		60.50 ± 17.0	
End of the study (n=24)		24.63 ± 4.1			24.75 ± 8.4		49.38 ± 3.5	
2 tailed p		0.001			0.004		0.000	
Post-hoc analysis stratified by A1C.								
	A1C < 7% < 53 mmol/mol		A1C ≥ 7% ≥ 53 mmol/mol	A1C < 7% < 53 mmol/mol	A1C ≥ 7% ≥ 53 mmol/mol	A1C < 7% < 53 mmol/mol		A1C ≥ 7% ≥ 53 mmol/mol
*Beginning of the study*	*31.50 (7.72) (n=10)*		*27.64 (3.79) (n=14)*	*33.70 (14.64) (n=10)*	*29.50 (12.8) (n=14)*	*65.20 (21.16) (n=10)*		*57.14 (13.13) (n=14)*
*End of the study*	*24.25 (4.14) (n=12)*		*25.00 (4.26) (n=12)*	*22.17 (6.95) (n=12)*	*27.33 (9.22) (n=12)*	*46.42 (10.6) (n=12)*		*52.33 (11.91) (n=12)*

Conventional therapy data represent 1st set of data (Beginning of the study), and AHCL represents second set of data (End of the study); n, sample size; Mean, average value of all participants; SD, standard deviation.

A post hoc breakdown comparison between children and adolescents with A1C < 7% (< 53 mmol/mol) versus children and adolescents with A1C ≥ 7% (≥ 53 mmol/mol) at the beginning and the end of the study also showed a reduction in fear of hypoglycemia with the use of AHCL in both < 7% (< 53 mmol/mol) and ≥ 7% (≥ 53 mmol/mol) (**Table 3**).

#### PAID problem areas in diabetes scale

The results show a statistically significant reduction in diabetes-related emotional distress from 19.3 ± 12.3 to 8.6 ± 8.3 (p>0.001) with the use of AHCL.

A post – hoc breakdown comparison children and adolescents with A1C < 7% (< 53 mmol/mol) versus children and adolescents with A1C ≥ 7% (≥ 53 mmol/mol) at the beginning of the study also shows a reduction in diabetes – related emotional distress with the use of AHCL in both < 7% (< 53 mmol/mol) and ≥ 7% (≥ 53 mmol/mol) (**Table 4**).

**Table 4:** Post – hoc comparison of average PAID Problem Areas in Diabetes Scale among children and adolescents with A1C < 7% (< 53 mmol/mol) versus children and adolescents with A1C ≥ 7% (≥ 53 mmol/mol).

PAID Problem Areas in Diabetes ScaleMean (SD)			
	A1C < 7% < 53 mmol/mol	A1C ≥ 7% ≥ 53 mmol/mol	All
Beginning of the study	18.00 (12.01) (n=10)	20.27 (12.79) (n=14)	19.32 (12.25) (n=24)
End of the study	9.79 (9.88) (n=12)	7.50 (6.48) (n=12)	8.65 (8.26) (n=24)

Conventional therapy data represent 1st set of data (Beginning of the study), and AHCL represents second set of data (End of the study); n, sample size; Mean, average value of all participants; SD, standard deviation.

#### WHO-5 five item measure of wellbeing

The average WHO-5 score of well-being was increased from the beginning of the study (68.17 ± 16.83) compared to the end of the study (80.50 ± 14.02) (p=0.03). The significance was not below the adjusted threshold off p<0.006 (p=0.03).

A post – hoc breakdown comparison between children and adolescents with A1C < 7% (< 53 mmol/mol) versus children and adolescents with A1C ≥ 7% (≥ 53 mmol/mol) at the beginning of the study also shows an increase in WHO-5 well-being score with the use of AHCL in both A1C < 7% (< 53 mmol/mol) and ≥ 7% (≥ 53 mmol/mol) subgroups (**Table 5**) with no statistically significant difference within the group with higher A1c at baseline (t (13) = -2.08, p =0.058, Cohen’s d (based on differences) with Hedges correction = 0.54 (medium/moderate size effect). The group with lower A1c at baseline shows a statistically significant increase in quality of life (t (9) = -3.74, p = 0.005, Cohen’s d (based on differences) with Hedges correction = 1.13 (very large/strong size effect).

**Table 5:** Post – hoc comparison of average World Health Organization – Five Well – Being score (WHO-5) among children and adolescents with A1C < 7% (< 53 mmol/mol) versus children and adolescents with A1C ≥ 7% (≥53 mmol/mol).

Organization-Five Well-Being Index (WHO-5) Mean (SD)			
	A1C < 7% < 53 mmol/mol	A1C ≥ 7% ≥ 53 mmol/mol	All
**Beginning of the study**	74.40 (14.39) (n=10)	63.71 (17.5) (n=14)	68.17 (16.83) (n=24)
**End of the study**	79.00 (16.72) (n=12)	82.00 (11.25) (n=12)	80.50 (14.02) (n=24)

Conventional therapy data represent 1st set of data (Beginning of the study), and AHCL represents second set of data (End of the study); n, sample size; Mean, average value of all participants; SD, standard deviation.

### Correlations among questionnaires’ results

In the A1C ≥ 7% (≥ 53 mmol/mol) subgroup of children and adolescents at the beginning of the study we observed a statistically significant negative correlation between PAID-20 and WHO-5 scores (r=0.546, p=0.043) indicating that increased diabetes-related emotional distress had a negative impact on the well-being of children and adolescents. We also observed a positive correlation between PAID-20 and CHFS total (r= 0.552, p = 0.041) indicating that diabetes-related emotional distress is correlated with fear of hypoglycemia. This was also observed in the < 7% (< 53 mmol/mol) subgroup of children and adolescents at the end of the study (r= 0.635, p= 0.027). There were no statistically significant correlations among other subgroups.

### Focus groups interviews

In the analysis of the interviews the baseline transcripts revealed 24 categories and 157 codes. The analysis of the qualitative data gathered after the introduction of AHCL generated 17 categories and 122 codes. Participants emphasized exhaustion with disease management, quality of sleep, and school related stress with dysglycemia.

Based on these results we confirmed that the burden of T1DM management was lower with the use of AHCL. We also confirmed that average well-being increased with the use AHCL. Additionally, the quality of life experienced by children and adolescents with T1DM using AHCL has increased (**Table 6**).

**Table 6:** Results: Focus groups interviews.

Themes	Beginning of the study (n=24)	End of the study (n=24)
**Quality of life**	• exhaustion disease management • spend a lot of time managing T1DM • more exhausted • more thinking about the disease • more stress • more fluctuations in blood glucose • more events of hyperglycaemia events • fear of hypoglycemia • a lot of thinking about the disease	• quality of life increased • less exhausted • more energy • less thinking about the disease • less stress • less involvement in disease management • spend more time in TIR • less fear of hypoglycemia • less fluctuations glucose • better managing of T1DM • better metabolic control • better quality of life
**Parents involvement**	• parents checking glucose levels • parents concern at night due to glucose fluctuations • parents measure glucose at night • parents control of managing of T1DM • parents are strongly involved in managing of T1DM	• positive parents experience with AHCL • less fear about glucose fluctuations • parents are less involved in managing of T1DM • parents trust of AHCL • better quality of sleep • experience of parents using AHCL was positive • less wake up at night • lower burden in managing of T1DM
**School environment**	• increase blood glucose during class • more hyperglycemia events at school • problems with managing of hyperglycemia - stress at school • confusion when participants have a hyperglycemia at school • harder concentrate on lessons	• fewer fluctuations in blood glucose • less hyperglycaemia events • better managing of hyperglycaemic events • better concentration in class • less thinking about the disease • better well-being • more energy in the school
**Quality of sleep**	• waking up at night • night glucose measurements • fear of hypoglycaemia at night	• better quality of sleep • less fear of hypoglycaemia at night • better quality of life

**Table 7:** Clinical and glycemic data.

	*Beginning of the study* (n=24)	*End of the study *(n=24)	*2-tailed repeated measures paired sample t test [p]*
Mean glucose mmol/L	8.55 ± 1.34 [6.80-11.58]	7.74 ± 0.42 [7.08-8.93]	0.002
TBR < 3.0 mmol/L [%]	0.84 ± 1.13 [0-4.32]	0.73 ± 0.79 [0.07-3.42]	0.577
TBR <3.9 mmol/L [%]	3.77 ± 3.69 [0-13.98]	3.00 ± 2.00 [0.78-10.18]	0.276
TIR (Time in range) [%]	68.22 ± 13.90 [40.22-88.63]	78.26 ± 6.29 [60.75-87.06]	0.000
TAR > 10 mmol/L [%]	28.00 ± 14.65 [7.53-59.78]	18.7 ± 5.43 [10.53-31.52]	0.001
TAR > 13.9 mmol/L [%]	7.92 ± 8.68 [0.35-31.20]	3.58 ± 2.71 [0.78-11.42]	0.006
SD of mean glucose mmol/L	3.06 ± 0.76 [2.18-4.92]	2.76 ± 0.45 [2.14-3.94]	0.011
CV of mean glucose [%]	35.7 ± 5.6 [28.93-50.79]	35.5 ± 4.4 [28.52-48.69]	0.856

Conventional therapy data represent the 1st set of data (Beginning of the study), and AHCL represents second set of data (End of the study); n, sample size, t-test for paired samples was used and 2 - tailed statistical significance. CV - coefficient of variation, SD – standard deviation.

The participants were more rested, worried less about their illness, and consequently had more time for other things. A notable improvement in the quality of sleep of both - participants and their parents were reported. The use of AHCL contributed to the increase in the quality of life. The use of AHCL was reported to bring less stress in disease management and was consequently less exhausting. The management of T1DM was easier, as reflected also in improved metabolic control.

### Metabolic control

The average value of A1C at the beginning of the study (8.55 ± 1.34% (69.9 ± 12.3 mmol/mol)) was significantly higher than at the end (7.74 ± 0.42% (61.1 ± 2.2 mmol/mol)) TIR differed significantly between the use of conventional therapy 68.22 ± 3.69% and AHCL 78.26 ± 6.29 (p<0.001). The TBR, did not differ significantly between the two types of treatment, neither for TBR <3.0 mmol/L (p=0.577), nor TBR <3.9 mmol/L (p=0.276). The Time above range (TAR) was significant less for the AHCL: TAR > 10 mmol/L (p=0.001) and TAR > 13.9 mmol/L (p=0.006) (**Table 7**). No severe hypoglycemia or diabetic ketoacidosis were reported.

## Conclusions

The standardized PROMs (C-HFS, PAID, WHO-5) were used to assess the observed benefits of using AHCL over a conventional therapy. All three scales were sufficiently reliable for the use as independent measures. Our results showed a significant decrease of Fear of Hypoglycemia, a reduction in diabetes-related emotional distress, and an increase in general well-being as reported by the participants. There was an overall rise in participants’ satisfaction with the use of AHCL as compared to the conventional therapy, less exhaustion, and a higher quality of life. Children and adolescents reported lower levels of fear, emotional distress, and increased well-being related to their T1DM. A recent clinical trial evaluating AHCL reports improved results from emotional burden scale, and behavioral burden, but no changes in distress or hypoglycemia confidence; it is of interest that satisfaction was greater in those who were more frequently in auto-mode (22). A group from Australia also showed improved glycemic control and improved diabetes treatment satisfaction as a possible consequence of reduced worry and increased trust in AHCL (23).

Our qualitative results indicated reduced exhaustion and increased sleep quality. Participants spent less time managing TIDM using the AHCL indicating reduced disease burden. Similarly, a study with a different AHCL also reports increased quality of life with AHCL usage (24).

Additionally, the quality of life experienced by children and adolescents with T1DM increased with the use of AHCL. This was accompanied by an increased satisfaction of their parents. Recently, data for a randomized controlled trial also demonstrated increase glucose monitoring satisfaction with the use of AHCL (22).

Our study showed that children and adolescents with T1DM had better metabolic control using AHCL. Different studies report that metabolic control with AHCL usage is significantly improved (25, 26). A recent trial shows that participants treated with AHCL had fewer episodes of hyperglycemia, and the TIR improved (27). The AHCL system can be used safely in children and adolescents with T1DM without episodes of severe hypoglycemia or diabetic ketoacidosis (24), as was also demonstrated in our study.

Different studies demonstrated that users of AHCL have less hypoglycemia (21, 25–33). In our study, the TBR was low already at the beginning of the study and remained low at the end of study. Noteworthy, a majority of participants were using CGM at baseline and approximately half of them were using PLGS or first generation HCL. Participants spent less time in TAR when treated with AHCL, as demonstrated in several other studies (30, 34).

In conclusion, patient reported outcome measures and qualitative assessment demonstrated decreased fear of hypoglycemia, less emotional distress, increased quality of life and reduced burden of disease management with the use of AHCL. These outcomes were related to improved measures of metabolic control.

## Limitations

Our study has limitations: a single center was included, and the sample size was relatively small. Additionally, the duration of the trial was limited.

## Implications/relevance for diabetes educators

Our study demonstrated novel data on the quality of life improvement in children and adolescent with T1DM. This new knowledge could complement our standardized diabetes education programs both at disease onset as well as our educational programs for therapy intensification, particularly when introducing AHCL.

## Data availability statement

The raw data supporting the conclusions of this article will be made available by the authors, without undue reservation.

## Ethics statement

The studies involving human participants were reviewed and approved by Slovenian National Medical Ethics Committee. Written informed consent to participate in this study was provided by the participants’ legal guardian/next of kin.

## Author contributions

AG, KD, NB, and TB contributed to the study concept and design. NB and TB supervised the study. AG, JS, BS, KD, JS, and NB collected data, JS performed statistical analyses. All authors participated in data analysis and interpretation. The manuscript was drafted by AG, NB, and TB, and reviewed by all authors. NB and TB are the guarantors of this work and, as such, had full access to all the data in the study and takes responsibility for the integrity of the data and the accuracy of the data analysis. All authors read and approved the final submitted version of the manuscript.

## Funding

The study was supported in part by the Slovenian Research Agency front # P3-0343, and University Medical Center Ljubljana grant # 20200234.

## Acknowledgments

We thank all participants and their parents, and the diabetes team, particularly the CDEs.

## Conflict of interest

TB served on advisory boards of Novo Nordisk, Sanofi, Eli Lilly, Boehringer, Medtronic, Indigo and DreaMed Diabetes; received honoraria for participating on the speaker’s bureaux of Eli Lilly, NovoNordisk, Medtronic, Abbott, Sanofi, Aventis, Astra Zeneca and Roche;and owns stock from DreamMed Diabetes. TB’s institution received research grant support and TB received travel and accommodation expenses in some cases, from Abbott, Medtronic, Novo Nordisk, GluSense, Sanofi, Sandoz and Zealand Pharma. KD served on advisory boards of Eli Lilly and Pfizer; received honoraria for participating on the speaker’s bureaux of Abbott, Medtronic and NovoNordisk. KD is a member of the European Commission Expert panel for Medical Devices for Endocrinology and Diabetes. NB received honoraria for participation on the speaker’s bureau of Medtronic and Roche.

The remaining authors declare that the research was conducted in the absence of any commercial or financial relationships that could be construed as a potential conflict of interest.

## Publisher’s note

All claims expressed in this article are solely those of the authors and do not necessarily represent those of their affiliated organizations, or those of the publisher, the editors and the reviewers. Any product that may be evaluated in this article, or claim that may be made by its manufacturer, is not guaranteed or endorsed by the publisher.
